# Molecular Tools for Metastatic Colorectal Cancer Characterization

**DOI:** 10.33696/immunology.2.067

**Published:** 2020

**Authors:** Radhashree Maitra, Elisha Fogel, Ruwan Parakrama, Sanjay Goel

**Affiliations:** 1 Montefiore Medical Center, 1695 Eastchester Road Bronx, New York 10461, USA; 2 Albert Einstein College of Medicine, 1300 Morris Park Ave, Bronx, NY 10461, USA; 3 Department of Biology, Yeshiva University, 500 West W 185th Street, New York NY, 10033, USA

## Abstract

In our recent publication [1], we have explored at the molecular level the consequences of reovirus administration to patients with *KRAS* mutated colorectal cancer (CRC). This was the first reported study where transcriptome assay was performed on *KRAS* mutated CRC patients receiving reovirus (pelareorep) therapy. Using peripheral mononuclear cells as a tumor surrogate, we have identified several hundred genes that were significantly altered in a transcriptome assay of patients receiving pelareorep serving as their own controls (pre and post reovirus administration) and compared to untreated controls [2]. We focused primarily on 884 immune related genes and published the data for genes with significance probability of 0.001 (1 in thousand for a perfect random occurrence). Samples were collected at 48 hours, day 8 and day 15 post reovirus administration and compared for dynamic gene expression alterations over time. Using PBMC we also performed flow cytometry, cytokine ELISA, immunohistochemistry, and determination of the expression level of CRC specific microRNA miR-29a-3p. Our data supports the therapeutic competence of reovirus and identifies the four major ways by which it exerts its antitumor effects.

## Commentary

The American Cancer Society estimates there will be 156,540 new diagnoses and 53,200 deaths from colorectal cancer (CRC) in the United States in 2020 [3,4]. Among the newly diagnosed individuals, approximately 40–45% will carry a mutation in *KRAS*, a GTPase that under normal circumstances serves as a signal transducer and as an oncogene when mutated [5,6]. This mutation precludes the *KRAS* mutant CRC patients from receiving treatment with anti-EGFR monoclonal antibodies, which have significant therapeutic benefits for CRC patients with the wild-type *KRAS* gene [7].

Analysis of metastases from patients that developed resistance showed the emergence of *KR*AS amplification and acquisition of secondary *KRAS* mutations. Thus, *KRAS* mutations have been identified as frequent drivers of innate and acquired anti-EGFR resistance and USDFA guidelines restrict the administration of anti-EGFR monoclonal antibody therapy only for *KRAS* WT (CRC) patients [8]. This group of mutant KRAS CRC patients currently has no alternate viable FDA approved treatment options available and constitutes a cohort of unfulfilled medical requirements.

Our study addressed the need for novel treatments options for *KRAS*-mutated CRC patients. Reovirus, a double-stranded (ds) RNA virus-induced cytotoxicity in *KRAS*-mutated tumors [9]. In CRC reovirus has shown significant therapeutic effect both as single agent as well as in combination with chemotherapy [7]. While reovirus presents itself as a promising therapeutic agent it has not yet achieved FDA approval as a single drug due to the antiviral immune response of the host. One approach to improve the efficacy of reovirus therapy is to use it in conjunction with approved chemotherapy or radiotherapy; this is currently in clinical trials [7]. An alternative approach can be to harness host mechanisms that improve virus delivery and produce a favorable environment for viral propagation. To achieve this, a detailed understanding of human-reovirus interactions is essential. Delineating the molecular mechanism of host-virus interactions and the subsequent downstream signaling cascade in CRC will provide information that can be translated into improving the therapeutic efficacy of the virus, as well as to identify molecules that can serve as biomarkers of disease progression.

Our laboratory primarily focusses on molecular characterization of *KRAS* mutated CRC and one of the primary arms of the study includes the therapeutic intervention of biologic reovirus (pelareorep). In the current study we have proceeded with transcriptome analysis to understand the overall genetic alterations in CRC patients harboring *KRAS* mutation when treated with reovirus. The analysis included 8 (eight) patients of which 5 (five) were treated with reovirus; and 3 (three) were treated with background therapy of FOLFIRI and bevacizumab and served as control. With the limited availability of biological specimens, we have utilized peripheral blood mononuclear cells (PBMC) and serum samples as surrogate of the tumor from the previously defined patient cohort enrolled in phase 1 clinical trial (NCT01274624).

**Figure 1:**
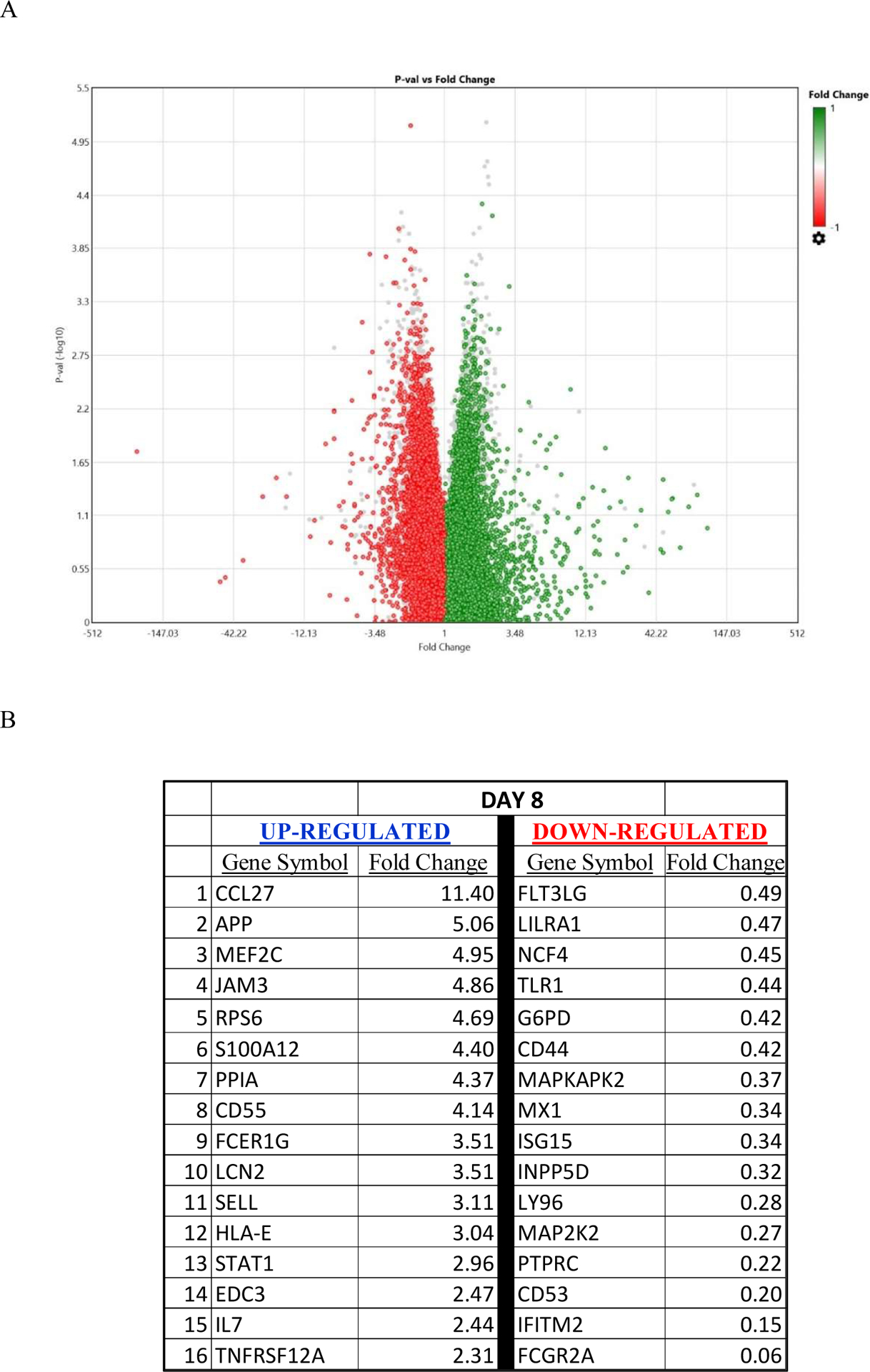
**A)** Pattern of alterations of gene expression pre and post (day 8) reovirus treatment. **B)** Representative data showing a list of 32 immune related genes that were significantly (p>0.05) altered at day 8 post pelareorep administration when compared to pretreatment conditions. 16 genes that were up-regulated are shown in blue and 16 that were down-regulated are shown in red along with the numerical fold change values.

The most complex endeavor is the accurate interpretation of the transcriptome data. Especially when the data involves the alterations of a few hundred genes. Figure 1 is the volcano plot showing the two thousand seven hundred and eighty transcripts with altered expression, red showing down regulation (at least halved) and green showing upregulation (at least doubled) between PRE (before REO) and day 8 of the experimental group.

Our analysis shows that the administration of reovirus results in immunomodulatory changes across transcript, protein, and cellular levels. Few notable changes include alterations in expression of several genes including *KRAS*, *VEGFA*, and *CXCR2*. A very interesting finding was an increase as high as 33-fold of mutant *KRAS* at 48 hours (p<0.001). It is intriguing how reovirus induces the expression of mutated *KRAS*. It is apparent that whether reoviral machinery induced the expression of *KRAS* or the cancer cell’s response to virus invasion is manifested by boosting up *KRAS* expression, it had overall favorable consequences involving cancer cell lysis and release of infective virions which are now capable of infecting neighboring cancer cells, thus generating a self-renewable therapeutic source. *In vitro* studies showed that reovirus causes preferential lysis of *KRAS* mutated CRC cells [10] and newly released virions of reovirus from a *KRAS* mutated CRC cells have greater degree of infectivity as compared to the one released from a *KRAS* wild type CRC cells [11]. The study further showed that reovirus infection induces mutant *KRAS* activity within the CRC cells [11]. Our *in vivo* human study confirmed similar characteristics of reovirus. In absence of reovirus infection such induction of *KRAS* activity in CRC cells would indicate rapid proliferation and subsequent progression of cancer. In furtherance of the findings we are investigating how this over-expression of *KRAS* is beneficial for viral propagation, a question that remains unanswered after 15 years of scientific investigation with reovirus and transformed cells.

VEGFA is considered an important growth factor contributing actively in angiogenesis and vasculogenic growth. It further advances endothelial cell proliferation, promotes cell migration, inhibits apoptosis, and induces permeabilization of blood vessels [12]. Our transcriptome analysis showed a twofold decrease in the expression of VEGFA at day 8 post reovirus administration when compared to pre levels (p<0.001). Such downregulation was not observed in control patients. We are confident that reovirus negatively impacts angiogenesis and thus exhibits strong tumorostatic/tumoricidal effects. Furthermore, an additional analysis of genes that are up-regulated by 2-fold and down-regulated by 0.5-fold at a p-value <0.05 showed that VEGFA is reduced across 48 hours, day 8 and day 15 timepoints.

Another important finding of the study was the downregulation of G protein coupled receptor protein CXCR2 (chemokine receptor 2) at p<0.001 on day 15. CXCR2 is a ligand for Interleukin 8 which is a prominent anti-inflammatory cytokine [13]. Interestingly our cytokine ELISA study further validated this observation with down regulation in expression of IL8 cytokine at the protein level, supporting the fact that reovirus induces anti-inflammatory and anti-tumorigenic effects in *KRAS* mutated CRC cells.

When focusing only on the 884 immune related genes we found more than 100 genes transcripts were significantly upregulated (>2 fold; p<0.05) which included 8 transcripts that are significantly upregulated at all three time points. Several genes that are involved in immune stimulation were found to be upregulated and includes *FGCR2A, IFNAR1, STAT3, KLRD1, TAP1, CD244*. Furthermore, about 50 gene transcripts were also downregulated and many of them have been identified to have pro-tumorigenic properties. Currently we are in the process of dissecting the entire data focusing on various cancer related pathways and following the gene expression in a time course dependent manner. Our analysis showed that six major *KRAS* related pathways including PI3K pathway is significantly altered upon pelareorep administration. Similar to our observation with the CRC cell line [10], we have observed an induction of apoptosis in human subjects upon pelareorep administration (unpublished data) with an overexpression of caspase 8 and 9 and downregulation of *FASLG* and *TGF2*.

The study also included determination of cellular and exosome levels of CRC specific microRNA miR-29a-3p which showed significant reduction at all time points. This microRNA is known to be associated with decreased expression of the tumor suppressor *WWOX* gene. This gene functions synergistically with p53/TP53 to control stress-induced cell death and plays a crucial role in *TGFB1* signaling and TGFB1-mediated cell death [14]. A comprehensive analysis of a panel of 25 cytokines showed increases in serum anti-tumorigenic cytokines GM-CSF, IL-15 and IL-12p40 and IL-12p70; reductions in serum pro-tumorigenic cytokines VEGF, RANTES and IL-8. Finally flow cytometry analysis indicated immune maturation of antigen presenting dendritic cells and activation of cellular immunity with increased number of matured CD4^+^ and CD8^+^ T-cells. Immunohistochemistry of core biopsy samples when compared to primary resection confirmed increased infiltration of T lymphocytes and significant increase in granzyme expression upon reovirus administration. The tumor morphology was altered with atrophy of tumor cells in discrete islands with strong staining for granzyme B and surrounded by fibrous connective tissues, a phenomenon commonly observed post tumor regression.

**Figure 2:**
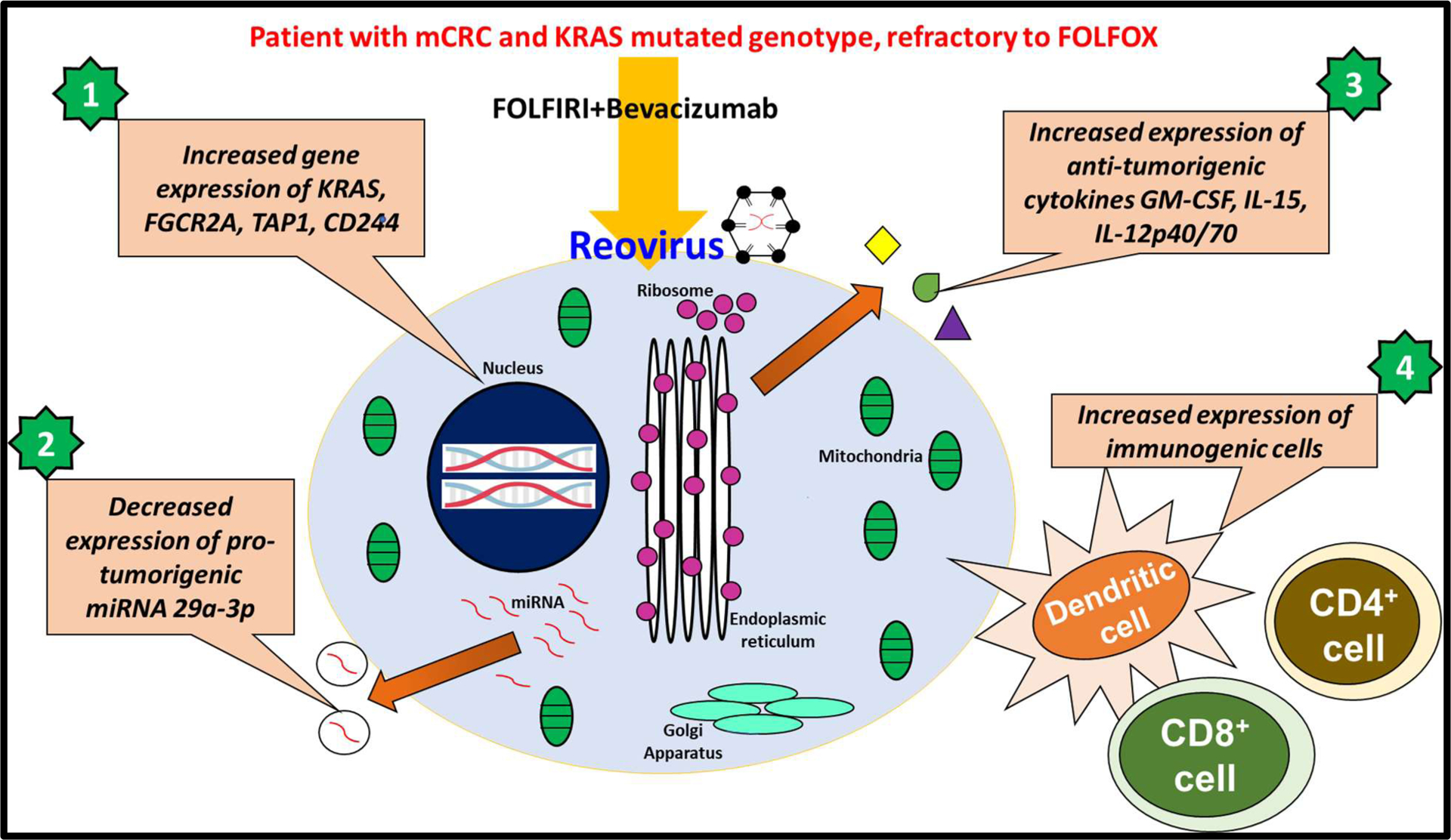
The illustration depicts the four major ways in which reovirus impacts the colorectal cancer cells upon infections. Each event can be corelated to anti- tumorigenic effect of reovirus.

These observations have prompted us to conclude that reovirus is a viable therapeutic agent for *KRAS* mutated CRC. It is a multipotent biological agent that induces anti-tumorigenic activities by four major ways as illustrated in Figure 2. These findings lend valuable insight into the potential of reovirus as an adjuvant when administered with established chemotherapy regimens in patients with *KRAS*-mutated metastatic mCRC. The findings warrant further study of reovirus in other cancers.
